# ASK1-ER stress pathway-mediated fibrotic-EV release contributes to the interaction of alveolar epithelial cells and lung fibroblasts to promote mechanical ventilation-induced pulmonary fibrosis

**DOI:** 10.1038/s12276-022-00901-1

**Published:** 2022-12-06

**Authors:** Ri Tang, Shuya Mei, Qiaoyi Xu, Jinhua Feng, Yang Zhou, Shunpeng Xing, Zhengyu He, Yuan Gao

**Affiliations:** Department of Critical Care Medicine, Renji Hospital, School of Medicine, Shanghai Jiaotong University, 200127 Shanghai, China

**Keywords:** Diseases, Molecular biology

## Abstract

Recent clinical research has revealed that mechanical ventilation (MV) can initiate pulmonary fibrosis and induce mechanical ventilation-induced pulmonary fibrosis (MVPF). However, the underlying mechanism remains largely uncharacterized. Based on a mouse model of MVPF and an alveolar epithelial cell cyclic strain model, the present study explores the possible mechanism of MVPF. Single-cell RNA-sequencing and EV RNA-sequencing analysis revealed that MV promoted apoptosis signal-regulating kinase 1 (ASK1)-mediated endoplasmic reticulum (ER) stress pathway activation and extracellular vesicle (EV) release from alveolar epithelial cells. Furthermore, the ASK1-ER stress pathway was shown to mediate mechanical stretch (MS)- or MV-induced EV release and lung fibroblast activation in vivo and in vitro. These processes were suppressed by ER stress inhibitors or by silencing ASK1 with ASK1- short hairpin RNA (shRNA). In addition, MVPF was suppressed by inhibiting ASK1 and ER stress in vivo. Therefore, the present study demonstrates that ASK1-ER stress pathway-mediated fibrotic-EV release from alveolar epithelial cells contributes to fibroblast activation and the initiation of pulmonary fibrosis during MV. The inhibited release of EVs targeting the ASK1-ER stress pathway might be a promising treatment strategy for MVPF.

## Lung disease: stress response pathway linked to ventilator-induced fibrosis

Cells that line airway sacs in the lungs react to the mechanical strain imposed by ventilators by activating a stress response pathway that may be targetable to prevent or treat ventilator-induced pulmonary fibrosis. A team from Ren Ji Hospital, Shanghai Jiao Tong University School of Medicine in China led by Zhengyu He and Yuan Gao profiled gene expression patterns in mice that developed lung fibrosis following mechanical ventilation and in cultured airway sac cells exposed to deforming tension in the laboratory. The researchers showed that the physical strain on the lung tissue activates a key cellular stress response pathway mediated by a protein called ASK1, which is associated with various stress responses. Lung alveolar cells consequently secrete small vesicles containing signaling molecules that prompt another population of cells, lung fibroblasts, to deposit collagen, a process that drives lung fibrosis.

## Introduction

In the development of acute respiratory distress syndrome (ARDS), pulmonary fibrosis (PF) plays an important pathological role. As a preferred treatment for respiratory failure, mechanical ventilation (MV) is widely used in the treatment of ARDS or PF. However, MV can cause ventilator-induced lung injury (VILI) and aggravate ARDS^1^. Furthermore, recent studies have revealed that MV can initiate lung fibrosis and induce mechanical ventilation-induced pulmonary fibrosis (MVPF)^2,3^. However, the underlying mechanism remains largely uncharacterized.

In recent years, the influence of the pathological microenvironment on cell dysfunction has been widely recognized as essential to the pathogenesis of PF^4^. In addition to the effect of traditional biological or chemical factors on cell dysfunction, the effect of physical microenvironment changes caused by mechanical stretch (MS) on cellular dysfunction has recently been identified as a novel physical factor associated with MVPF pathogenesis^3,5^. An increasing number of studies have revealed that different types of cells interact with each other through extracellular vesicles (EVs) and thus form a cellular regulatory network in lung tissue to promote the progression of PF^6^. However, whether the EV-mediated interaction between alveolar epithelial cells and lung fibroblasts under MS-induced microenvironment alteration plays a substantial role during the progression of MVPF is unclear.

As a result of our single-cell analysis, MVPF was found to be associated with an increase in the expression of genes involved in ER stress in epithelial cells. EV bulk RNA sequencing implied that EV function was associated with the progression of pulmonary fibrosis. Therefore, we hypothesized that MV-induced fibrotic-EVs release from lung epithelial cells to facilitate the activation of lung fibroblasts and development of MVPF. Previous studies have shown that apoptosis signal-regulating kinase 1 (ASK1) is related to the ER stress pathway^7^, but the role of these processes in MVPF remains unclear.

This study is based on a mouse model of MVPF and an alveolar epithelial cell cyclic strain model, and aims to clarify the role of the ASK1-ER stress pathway in crosstalk between epithelial cells and fibroblasts in MVPF.

## Materials and methods

### Ethics statement and animals

C57BL/6 male mice (6–8 weeks old; 18–27 g) were acquired from Shanghai SLAC Laboratory Animal, China. The animals were maintained in a controlled environment with a temperature of 22–24 °C, a 12 h light/dark cycle, and free access to food and water. The Animal Care and Use Committee of Ren Ji Hospital, Shanghai Jiao Tong University School of Medicine approved all the experiments.

### Cell lines and culture

The human lung fibroblast MRC-5 cell line and the murine alveolar epithelial cell line TC-1 were acquired from the Cell Bank of the Chinese Academy of Sciences (Shanghai, China).

The MRC-5 cells were cultured in minimum essential medium (MEM, HyClone, USA) supplemented with 10% fetal bovine serum (FBS, Gibco, USA), 100 IU/ml penicillin, and 100 IU/ml streptomycin, while the TC-1 cells were cultured in RMPI 1640 medium (1640, Gibco, USA) supplemented with 10% FBS, 100 IU/ml penicillin, and 100 IU/ml streptomycin. To remove EVs from the FBS used in the cell cultures, the medium was ultracentrifuged at 100,000 × *g* for 70 min.

### MV model and animal procedures

C57BL/6 male mice (6–8 weeks old; 18–27 g) were anesthetized with an intraperitoneal injection of 200 mg/kg ketamine and 10 mg/kg xylazine and randomly allocated to sham and MV group. The mice in the MV group were mechanically ventilated for 2 h using FiO2 0.21, VT 20 ml/kg, and RR 70 breaths/min^8^, while the sham group maintained spontaneous breathing after intubation.

An intratracheal injection of ASK1 adeno-associated virus (AAV) to knockdown ASK1 expression in pulmonary tissue and intraperitoneal injection of the ER stress inhibitor 4-phenylbutyric acid (4-PBA) were administered to inhabit ER stress (Supplementary Fig. 1a). Observation was performed over a 7-day period following intubation of the animals, which had with free access to food and water, in the animal facility.

To obtain samples of bronchoalveolar lavage fluid (BALF), 20 G intratracheal cannulas were used after the experiments were completed. To recover the BALF, the right lung was washed with 500 μL of cold PBS and then collected and centrifuged at 3000 × *g* for 10 mins. The total protein level of the BALF was measured, or the BALF was used for EV isolation. After BALF was collected, the sample from right lung tissue was used for western blotting (WB), PCR, and flow cytometry. Histopathology, immunohistochemistry, and transmission electron microscopy (TEM) were performed on left lung tissue, which was fixed in paraformaldehyde.

### ASK1 inhibition by knockdown AAV transfection

Adeno-associated viruses (AAVs) were acquired from Genomeditech Co., Ltd. (Shanghai, China), and virus infection constructed stable TC-1 cell lines.

Prior to intubation, mice received AAVs expressing ASK1-short hairpin RNA (shRNA), as well as a vector control, the lungs of mice were intranasally injected with 50 μl of PBS containing 1 × 10^12^ μg of shRNA per mouse for 4 weeks.

C57BL/6 male mice (6–8 weeks old; 18–27 g) were randomly assigned to sham, ASK1-shRNA, MV, and MV + ASK1-shRNA groups. Mice in the MV and MV + ASK1-shRNA groups received MV treatment for 2 h.

### ER stress inhibition by 4-phenylbutyric acid (4-PBA)

4-PBA (20 mg/kg in corn oil) is an ER stress inhibitor^9^. Before intubation, 4-PBA was administered intraperitoneally to mice once per day for three consecutive days.

Male C57BL/6 male mice (6–8 weeks old; 18–27 g) were randomly assigned to the sham, 4-PBA, MV, and MV + 4-PBA groups. Mice in the MV and MV + 4-PBA groups received MV treatment for 2 h.

### Mechanical stretch

The MS method was used to mimic the effects of MV in vivo on TC-1 cells. MS was applied to TC-1 cells using a Flexcell FX4000 AFC-CTL cyclic stress unit (Dunn Labortechnik, Asbach, Germany) using 20% elongation (1 Hz) over 24 h.

After the MS was completed, the cell culture medium was collected for EV isolation, and TC-1 cells were harvested for WB, flow cytometry, and transmission electron microscopy.

### Treatment of cells with ASK1 knockdown

To knock down ASK1, TC-1 cells were infected with AAVs. For viral infection experiments, 5E8TU/mL of Ad5 was used. Confluent cells were randomly assigned to sham, ASK1-shRNA, MS, and MS + ASK1-shRNA groups. The MS and MS + ASK1-shRNA cells were treated via MS for 24 h.

### Treatment of cells with 4-PBA

TC-1 cells were randomly assigned to sham, 4-PBA, MS, and MS + 4-PBA groups. Cells in the sham group received PBS treatment as a control; cells in the 4-PBA group were cultured in the medium containing 5 mM 4-PBA (Selleck, USA) for 2 h; cells in the MS group received MS treatment; cells in the MS + 4-PBA group were cultured in the medium containing 5 mM 4-PBA (Selleck, USA) for 2 h and then treated with MS for 24 h.

### Single-cell analysis

We used single-cell RNA-Seq to analyze mouse lung cells from the sham (*n* = 3) and MV groups (*n* = 3). Gene expression data have been deposited into the SRA database at the NCBI with accession number SUB11878630. Rigel S2 (Countstar, China) was used to count the cells and determine the cell viability in each sample. The Chromium Single Cell 3′ V2 Chemistry Library Kit, Gel Bead & Multiplex Kit, and Chip Kit from 10x Genomics (Biomarker Technologies, China) were used to generate the single-cell libraries. To generate gel bead-in-emulsions (GEMs), cellular suspensions were loaded onto a chromium controller (10x Genomics, Pleasanton). According to the manufacturer’s instructions, barcoded sequencing libraries were generated using Chromium Single Cell 3′ Reagent Kit v3.1 (10x Genomics, Pleasanton). Each sample was sequenced using paired-end sequencing in one lane of a NovaSeq 6000, and each sequence was 150 nt after library preparation. Using the 10x Genomics Cell Ranger pipeline (https://support.10xgenomics.com/single-cell-gene-expression/software/downloads/latest), raw reads were processed using the mm10 as a reference. With Cell Ranger, single cells were clustered, marker genes were identified, and unique molecular identifiers (UMI) can bewere exported. To carry out further analysis, the Seurat R package (2.2) was used. The majority of Seurat analyses were performed with default parameters.

### Bulk RNA sequence analysis

We used RNA sequencing to analyze mRNA in EVs isolated from the sham and MV groups. Gene expression data have been deposited into the SRA database at the NCBI with accession number PRJNA 867324. Total RNA was isolated using TRIzol reagent (Takara Biotechnology, Japan) following the manufacturer’s instructions. Using a Ribo-Zero rRNA Removal Kit (Epicenter, USA), we obtained 1.5 μg of RNA from each sample, and this RNA was used as the input material for rRNA removal. For each sample, index codes were added to the sequencing libraries created using the NEBNext Ultra Directional RNA Library Prep Kit for Illumina (NEB, USA) according to the manufacturer’s recommendations. The index-coded samples were clustered using a cBot Cluster Generation System with TruSeq PE Cluster Kitv3-cBot-HS (Illumina, USA). An Illumina platform was used to sequence the preparations from the library and generate reads after cluster generation. An analysis of the FASTQ sequence data was performed using the BWA–Bowtie–Cufflinks workflow. Specifically, BWA and Bowtie software were used to map sequence reads to the GRCm38/mm10 assembly. The DESeq2 R package (1.34.0) was used to analyze differential gene expression between the MV and sham groups. A gene was considered to be a differentially expressed gene by DESeq when the adjusted P value was less than 0.01 and the absolute log2 (fold change) was greater than 1. The ClusterProfiler R package (4.2.2) was used to perform Gene Ontology enrichment analyses of the differentially expressed genes (DEGs).

### Pulmonary histopathology

Lung tissue was fixed with 4% paraformaldehyde overnight and then dehydrated and embedded in paraffin. A 5-μm-thick section of lung was stained with hematoxylin and eosin to evaluate lung morphological changes, and Masson’s trichrome stain was used to identify collagen deposition.

### Immunofluorescence staining

Formalin-fixed, paraffin Section (4 μm) of pulmonary tissues fixed with 4% PFA and permeabilized with 0.25% Triton X100 were stained with primary antibodies (details in Supplementary Table 1). Alexa Fluor 488-conjugated anti-rabbit IgG (Invitrogen, CA) and Alexa Fluor 594-conjugated anti-rabbit IgG (Invitrogen, CA) were used as secondary antibodies. DAPI (Santa Cruz Biotechnology, Germany) was used to detect nuclei. A Leica TCS SP2 confocal laser scanning microscope was used to capture images.

### Flow cytometry

For collection, TC-1 cells were washed with PBS, then trypsin (Beyotime, China) was used. The cells were incubated with Fix/Permed by in a Transcription Factor Buffer Set (BD, 562574) at room temperature in the dark for 15 min.

Subsequently, the cells were incubated with p-ASK1 (Abcam, ab278785) for 30 min at room temperature in the dark for 30 min. BD Biosciences FACSVerseTM was used to test all the samples, and FlowJo 10.4 software was used to analyze the results.

### Transmission electron microscopy

To perform transmission electron microscopy, formalin-fixed, paraffin Section (4 μm thick) of pulmonary tissues were harvested and washed with 0.1 M phosphate buffer (PB) and then fixed with 2% glutaraldehyde and 2% paraformaldehyde in 0.1 M PB for 3 min and postfixed with 1% osmic acid for 1 h. After dehydrating with ethanol, washing with propylene oxide, and embedding in epoxy resin, the samples were dehydrated in ethanol. Semithin and ultrathin sections previously cut using a Reichert ultramicrotome and stained with lead citrate and uranyl acetate were viewed with a Hitachi HT7800 transmission electron microscope.

To conduct transmission electron microscopy of TC-1 cells, the cells were treated with 2.5% glutaraldehyde in 0.1 M PB, followed by 1% osmium tetroxide (OsO4) in 0.1 M PB. A common procedure was used to embed cells in Epon 812 after fixation. A Hitachi HT7800 transmission electron microscope was used to examine specimens sliced with a 70-lm Ultracut Reichert–Jung ultramicrotome and stained with uranyl acetate and lead citrate.

### Total protein extraction and BCA

Lung tissues, cells, or EVs were lysed by RIPA lysis buffer (Epizyme, China) with 1% phenyl methyl sulfonyl fluoride, a protease inhibitor cocktail, and a phosphatase inhibitor cocktail (Epizyme, China) for 15 min on ice. After centrifugation at 15,000×*g* for 15 min at 4 °C, the concentration of protein supernatant was determined using the BCA method and a BCA protein assay kit (Epizyme, China).

### Western blot analysis

Sodium dodecyl sulfate‒polyacrylamide gel electrophoresis was used to separate proteins, which were then transferred to polyvinylidene difluoride membranes. The primary antibodies listed in Supplementary Table 1 and the appropriate HRP-linked anti-rabbit IgG secondary antibodies (CST, 7074 S) were incubated with the membranes. An enhanced chemiluminescent substrate kit (Vazyme, China) was used to detect the blots with Image LabTM software (Bio–Rad, USA).

### Quantitative real-time-PCR (qRT‒PCR)

In accordance with the manufacturer’s instructions, total RNA was isolated from lung tissue or cells using an RNA purification kit (EZ Bioscience, USA). Prime Script RT Master Mix (Takara, China) was used for complementary DNA synthesis. iTaq Universal SYBR Green Supermix (Bio–Rad, Hercules, CA, USA) was used for real-time PCR on a Light Cycler 480 real-time PCR system (Roche, USA). For each sample, cDNA amounts were normalized to the amount of GAPDH. The primers were as follows: GAPDH forward, 5′-AGGTCGGTGTGAACGGATTTG-3′, and reverse, 5′-GGGGTCGTTGATGGCAACA-3′; and ASK1 forward, 5′-TTTGTTTCGTGAGACTGCGTACC-3′, and reverse, 5′-AGA CACTTGGGCACACTACACA-3′. Furthermore, the 2−ΔΔCt method was used to calculate relative expression levels.

### EV isolation, characterization, and labeling

The supernatant from BALF was collected after sequential centrifugation at 500 × g, 2500 × g, and 12,000 × g. Isolated pellets were resuspended in 20 µL of PBS and stored at 4 °C and used later after ultracentrifugation at 100,000 × *g* for 70 min.

Samples were placed on a carbon-coated copper mesh for 90 s before being stained with uranyl acetate dye solution for 30 s and visualized under a transmission electron microscope. NanoSight NS300 (Malvern Panalytica, UK) was used to measure the diameter of the EVs based on a nanoparticle tracking analysis (NTA). WB was performed to measure EV surface marker protein levels. In accordance with the manufacturer’s protocol, purified EVs were labeled with PKH-67 membrane dye (Sigma‒Aldrich, USA).

### Statistical analysis

The data are displayed as the mean ± SEM. Multiple comparisons with group and time factors were analyzed using a two-way analysis of variance followed by Bonferroni test. For in vitro experiments, a nonparametric test was performed to compare cells with and without stretch. Differences between variables were considered to be significant when the *p* value was <0.05 according to GraphPad Prism 9 (GraphPad Software Inc, CA).

## Results

### MV-induced pulmonary fibrosis was accompanied by fibrotic MV-EV release

**Fig. 1:**
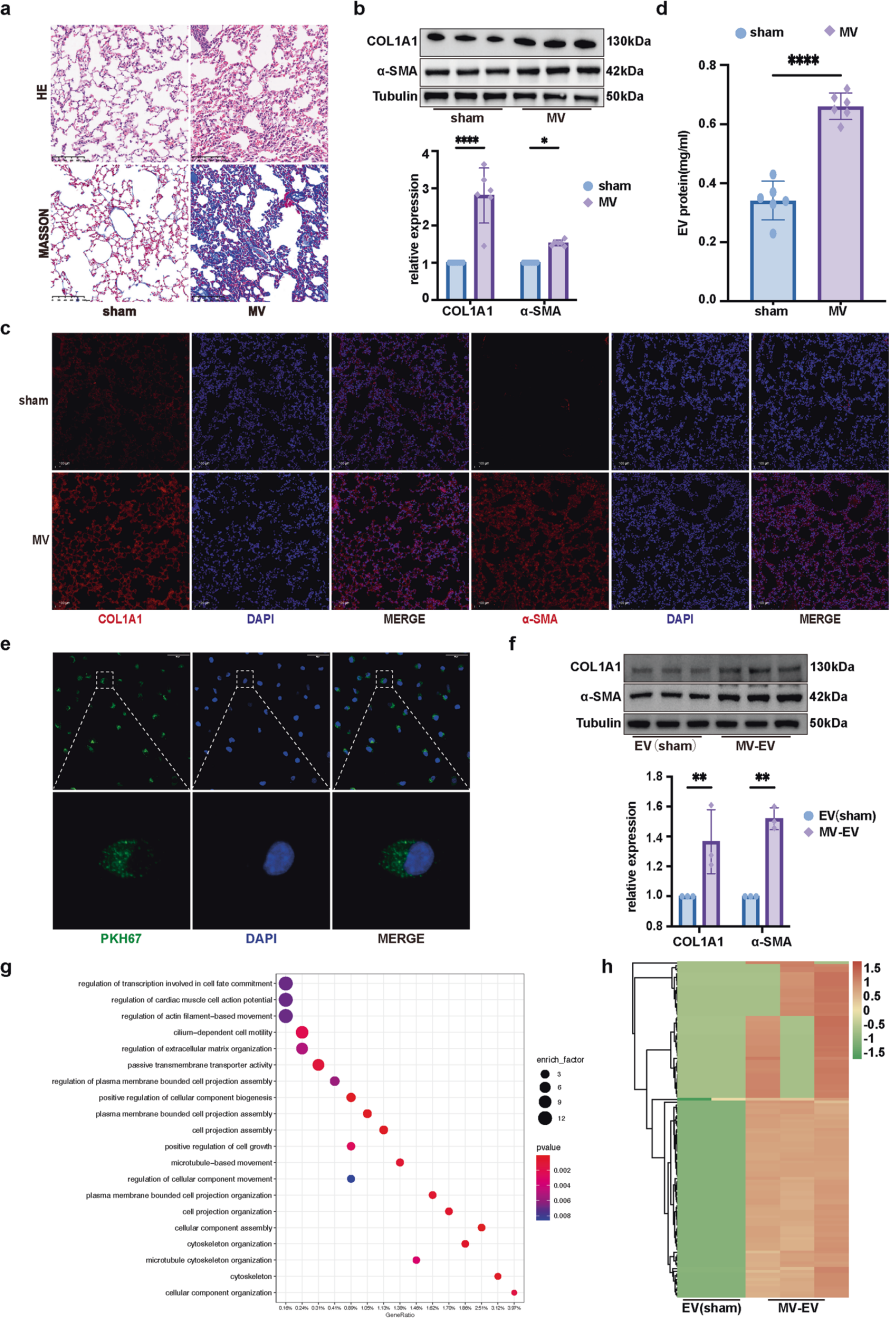
MV-induced pulmonary fibrosis was accompanied by fibrotic MV-EV release. **a** Lung injury was assessed by hematoxylin and eosin staining. Collagen deposition was assessed with Masson’s trichrome staining. Original magnification ×200. Scale bars correspond to 100 μm (*n* = 6). **b** Fibrosis was also quantified by the determination of collagen-I α1 (COL1A1) and α smooth muscle actin (α-SMA) levels in lung tissues by western blotting (WB). Relative intensity of the protein bands of COL1A1 and α-SMA compared to that of Tubulin as determined by densitometry is displayed in bar graphs (*n* = 6). **c** Lung tissues were stained with fluorophore-labeled antibodies against COL1A1 and α-SMA (Alexa Fluor 594, red). 4’,6-Diamidino-2-phenylindole (DAPI) stain was used to detect nuclei (blue). Original magnification ×200. Scale bars correspond to 100 μm (*n* = 6). **d** EVs were quantified by protein BCA. **e** Confocal microscopy shows the fluorescence of uptake of PKH-67-labeled EVs (Alexa Fluor 488, green) by MRC-5 cells. Original magnification ×400. Scale bars correspond to 50 μm (*n* = 3). **f** Protein expression of COL1A1 and α-SMA in MRC-5 cells was determined by WB. Relative intensity of the protein bands of COL1A1 and α-SMA compared to that of Tubulin as determined by densitometry is displayed in bar graphs (*n* = 3). **g** GO enrichment analysis of DEGs between the EV(sham) and MV-EV groups. **h** Heatmap of upregulated and downregulated fibrosis-associated gene mRNA expression in the EV (sham) and MV-EV groups. The data are expressed as the means ± SEMs. *p < 0.05, **p < 0.01, ***p < 0.001, ****p < 0.001.

To create the mouse model of MVPF in vivo, mice were observed 1 week after MV^10^. According to histological evaluation, pulmonary injury, interstitial leukocyte infiltration, alveolar edema, and hemorrhage were aggravated after MV compared to these measures in the sham group (Fig. 1a). Collagen deposits were increased in the pulmonary interstitium after MV, as evidenced by Masson staining (Fig. 1a). In addition, the protein levels of collagen α-1 type I (COL1A1) and α-smooth muscle actin in pulmonary tissue were increased (Fig. 1b). In accordance with the histological observations, immunofluorescence staining showed that MV increased the percentage of COL1A1+ and α-SMA+ cells compared with the percentage of these cells in the sham group (Fig. 1c).

EVs from the BALF of mice were isolated and purified. TEM and NTA were performed to determine the structure and size of the particles isolated from the BALF. The EV markers CD63, CD9, and Alix were detected by WB (Supplementary Fig. 1b). In addition, we found that concentration of EVs released was increased in the BALF after MV; these EVs were named MV-induced EVs (MV-EVs) (Fig. 1d). Then, the MRC-5 cell line was used as the target cells to evaluate the functions of the MV-EVs. After the MRC-5 cells were treated with MV-EVs (50 μg of EV protein/sample), PKH-67-labeled EVs were found to be taken up by MRC-5 cells (Fig. 1e). Increased protein expression of COL1A1 and α-SMA was apparent in the MV-EV group (Fig. 1f). Furthermore, consistent with the WB results, immunofluorescence staining showed that the MV-EV treatment of MRC-5 cells led to a significant increase in the percentage of COL1A1+ and α-SMA+ cells (Supplementary Fig. 1d).

To determine the mechanism by which MV-EVs promote fibrotic effects, we used EV bulk RNA sequencing. A heatmap of DEGs between the sham-EV and MV-EV is shown in Supplementary Fig. 1c. A GO enrichment analysis of the DEGs showed the fibrotic effect of the MV-EVs (Fig. 1g). Notably, the fibrosis-related mRNA levels were significantly increased in the MV-EV (Fig. 1h).

Therefore, it is reasonable to speculate that MV-induced fibrotic-EV release might be essential to MVPF.

### MS-induced ER stress-mediated fibrotic MS-EV release to promote lung fibroblast activation

**Fig. 2:**
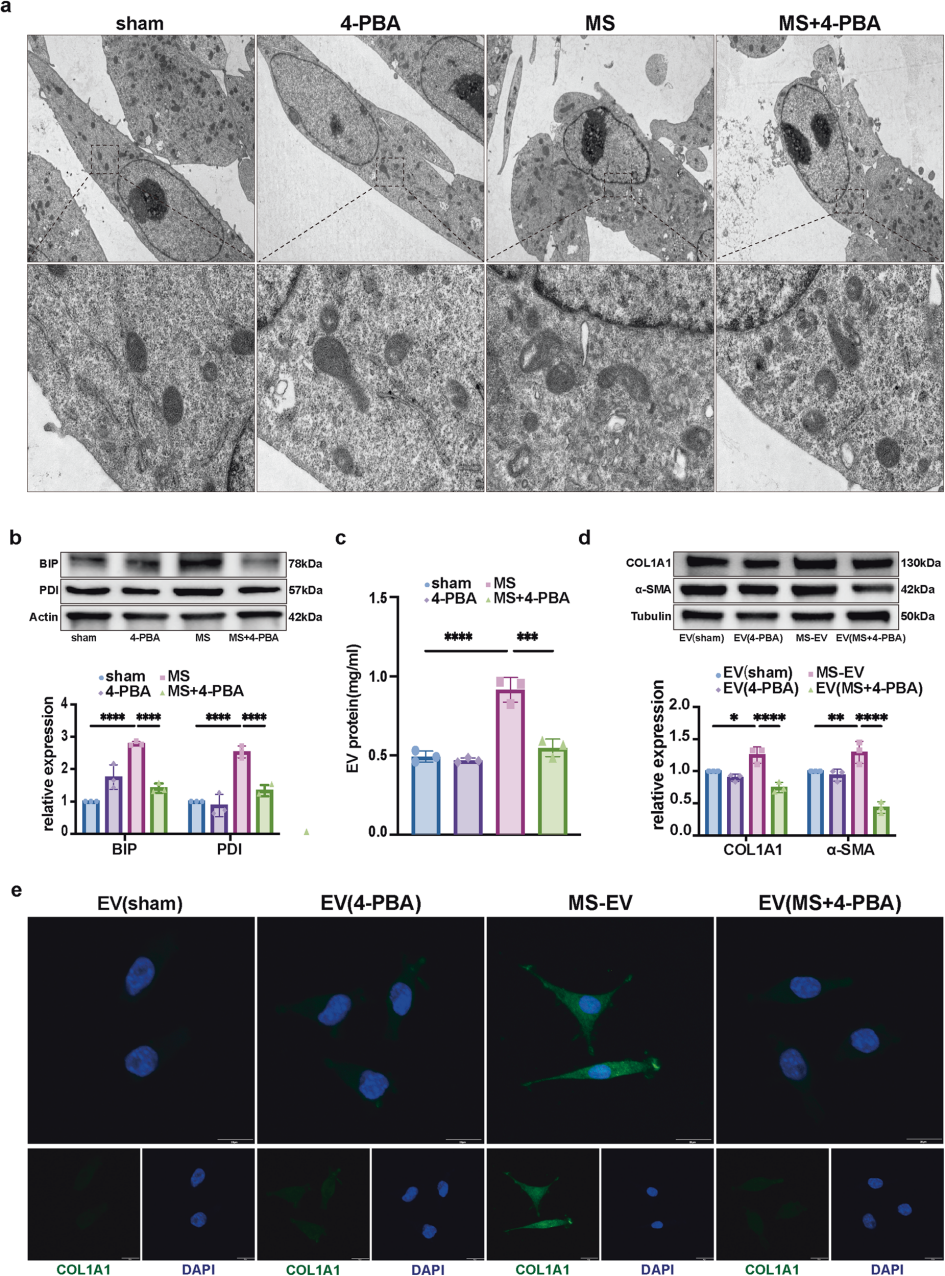
MS-induced ER stress-mediated fibrotic MS-EV release to promote lung fibroblast activation. **a** Representative transmission electron microscopy (TEM) images showing the ER in TC-1 cells (*n* = 3). **b** Protein expression of BIP and PDI in TC-1 cells as determined by WB. Relative intensity of the protein bands of BIP and PDI compared to that of Actin as determined by densitometry is displayed in bar graphs (*n* = 3). **c** EVs isolated from the cell supernatants of TC-1 cells were quantified by protein BCA (*n* = 3). **d** Protein expression of COL1A1 and α-SMA in MRC-5 cells was determined by WB. Relative intensity of the protein bands of COL1A1 and α-SMA compared to that of Tubulin as determined by densitometry is displayed in bar graphs (*n* = 3). **e** MRC-5 cells were stained with fluorophore-labeled antibodies against COL1A1 and α-SMA (Alexa Fluor 488, green). 4’,6-Diamidino-2-phenylindole (DAPI) stain was used to detect nuclei (blue). Original magnification ×800. Scale bars correspond to 20 μm (*n* = 3). The data are expressed as the means ± SEMs. *p < 0.05, **p < 0.01, ***p < 0.001, ****p < 0.001.

Recent studies have shown that ER stress can promote EV secretion in breast cancer cells^11^. Constituting the largest population of structural cells in lung tissues and the cells initially damaged by MV, alveolar epithelial cells were found to be the primary cells secreting EVs in lung tissues during MV. Whether this process is related to ER stress was unclear. Therefore, ER stress-related protein levels in TC-1 cells were measured after MS. As shown in Fig. 2a, the ultrastructure of the ER in the sham group showed normal morphology, while a significantly dilated ER was found in the MS group. The protein levels of the ER stress-related proteins BIP and PDI were increased significantly in the MS group (Fig. 2b).

Moreover, an increase in the concentration of EVs released was detected in the cell supernatant following MS which was named MS-induced EV (MS-EV) (Fig. 2c). After MRC-5 cells were treated with MS-EVs (50 μg of EV protein/sample), COL1A1 and α-SMA expression increased apparently in the MS-EV group (Fig. 2d). Furthermore, consistent with the WB results, immunofluorescence staining showed that MS-EV treatment of MRC-5 cells significantly increased the percentage of COL1A1+ (Fig. 2e) and α-SMA+ cells (Supplementary Fig. 1e).

To explore whether ER stress alters the release of EVs and the activation of lung fibroblasts, 4-PBA was used to reduce ER stress. The previously described outcomes were attenuated by pretreatment with 4-PBA before MS (Fig. 2a–e). 4-PBA pretreatment markedly reduced the EV release and alleviated the activation of MRC-5 cells.

Taken together, these findings suggested that MS-induced ER stress promoted MS-EV release, which might be partially responsible for lung fibroblast activation in vitro.

### MS-induced ASK1 activation mediated ER stress and fibrotic MS-EV release to promote lung fibroblast activation

**Fig. 3:**
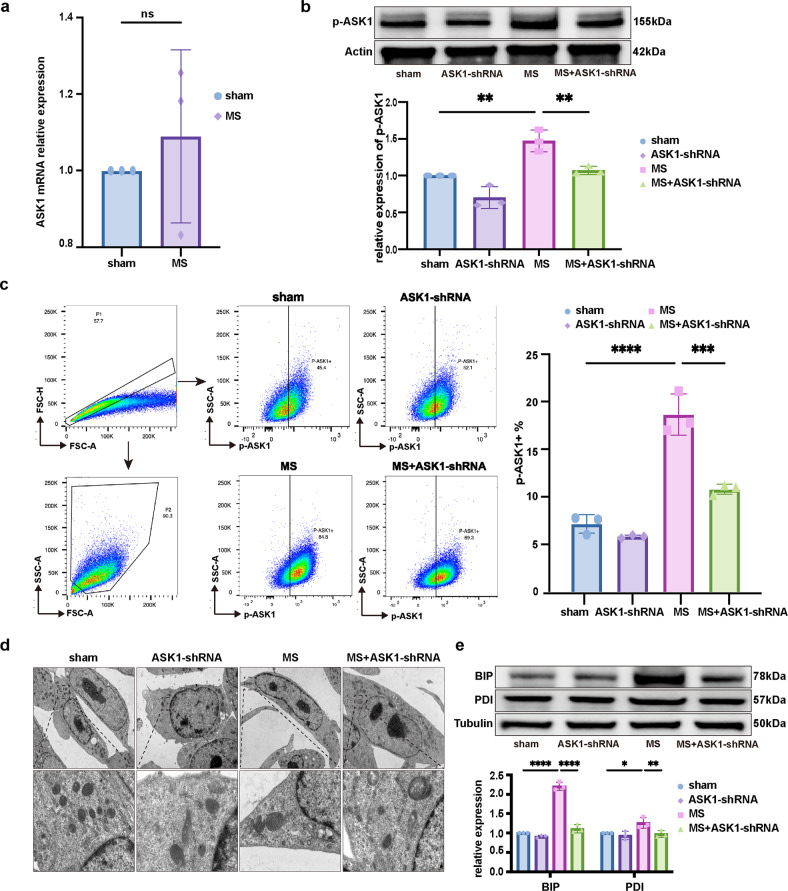
MS-induced ASK1 activation mediated ER stress in alveolar epithelial cells. **a** Relative mRNA expression of ASK1 compared to that of GAPDH is displayed in bar graphs (*n* = 3). **b** Protein expression of p-ASK1 in TC-1 cells was determined by WB. Relative intensity of the protein bands of p-ASK1 compared to that of Actin as determined by densitometry is displayed in bar graphs (*n* = 3). **c** Flow cytometry was performed to measure the expression of p-ASK1 in TC-1 cells (*n* = 3). **d** Representative TEM images of the ER in TC-1 cells (*n* = 3). **e** Protein expression of BIP and PDI in TC-1 cells was determined by WB. Relative intensity of the protein bands of BIP and PDI compared to that of Tubulin as determined by densitometry is displayed in bar graphs (*n* = 3). The data are expressed as the means ± SEMs. *p < 0.05, **p < 0.01, ***p < 0.001, ****p < 0.001.

ASK1 has been shown to be involved in ER stress-dependent EV-induced liver fibrosis in vitro^7^. Therefore, we sought to determine whether ASK1 activation contributes to MS-induced ER stress and MS-EV release. The qRT‒PCR results indicated that the mRNA level of ASK1 was not changed in the MS group (Fig. 3a), while the protein level of phosphorylated-ASK1 (p-ASK1) was increased in TC-1 cells after MS (Fig. 3b). Flow cytometry also demonstrated that the percentage of p-ASK1+ cells was increased significantly after MS (Fig. 3c).

**Fig. 4:**
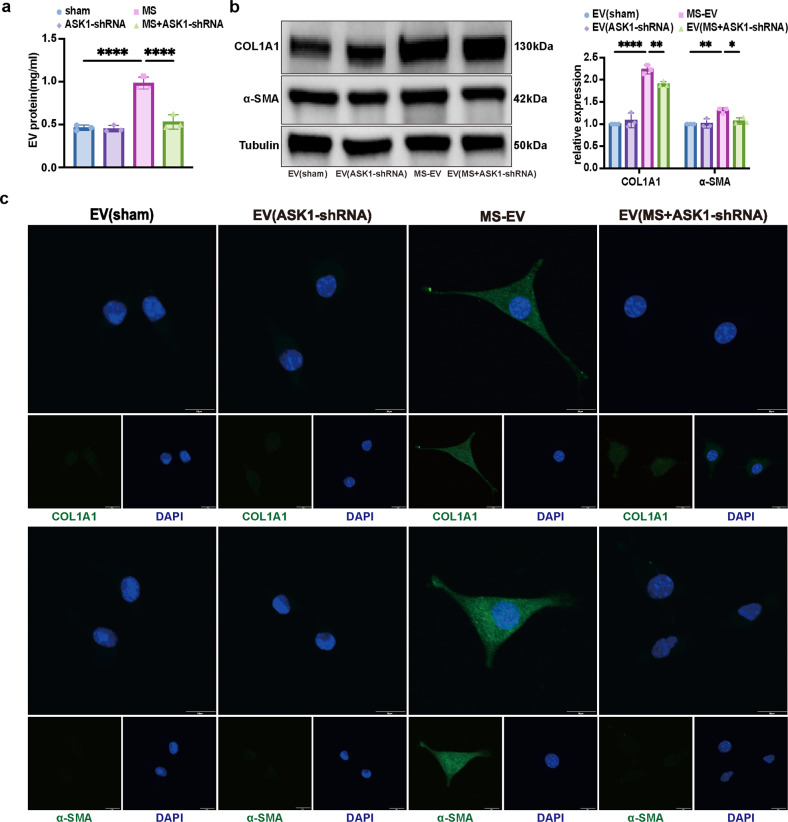
MS-induced ASK1 activation mediated fibrotic MS-EV release to promote lung fibroblast activation. **a** EVs isolated from the cell supernatants of TC-1 cells were quantified by protein BCA (*n* = 3). **b** Protein expression of COL1A1 and α-SMA in MRC-5 cells was determined by WB. Relative intensity of the protein bands of COL1A1 and α-SMA compared to that of Tubulin is displayed in bar graphs (*n* = 3). **c** MRC-5 cells were stained with fluorophore-labeled antibodies against COL1A1 and α-SMA (Alexa Fluor 488, green). 4’,6-Diamidino-2-phenylindole (DAPI) stain was used to detect nuclei (blue). Original magnification ×800. Scale bars correspond to 20 μm (*n* = 3). The data are expressed as the means ± SEMs. *p < 0.05, **p < 0.01, ***p < 0.001, ****p < 0.001.

To further investigate the role of ASK1, an ASK1-shRNA AAV was constructed to knock down ASK1 expression in TC-1 cells (Supplementary Fig. 1f). ASK1-shRNA transfection inhibited MS-induced ER stress in TC-1 cells, as indicated by the lower protein levels of BIP and PDI, and fewer ER ultrastructure changes were observed (Fig. 3d, e). As shown in Fig. 4a, ASK1-shRNA reduced the concentration of MS-EV released into the cell supernatant after MS. Furthermore, after treating MRC-5 cells with MS-EVs (50 μg EV protein/sample), upregulation of COL1A1 and α-SMA protein expression was detected by WB (Fig. 4b). This result was further confirmed in the MS-EV group via immunofluorescence assay (Fig. 4c). These processes were suppressed by ASK1 silencing with ASK1-shRNA (Fig. 4a–c).

Considering these results, we speculated that MS activated the ASK1-ER stress pathway to increase fibrotic MS-EV release and mediate the activation of lung fibroblasts.

### MV-induced ER stress-mediated fibrotic MV-EV release to promote lung fibroblast activation

**Fig. 5:**
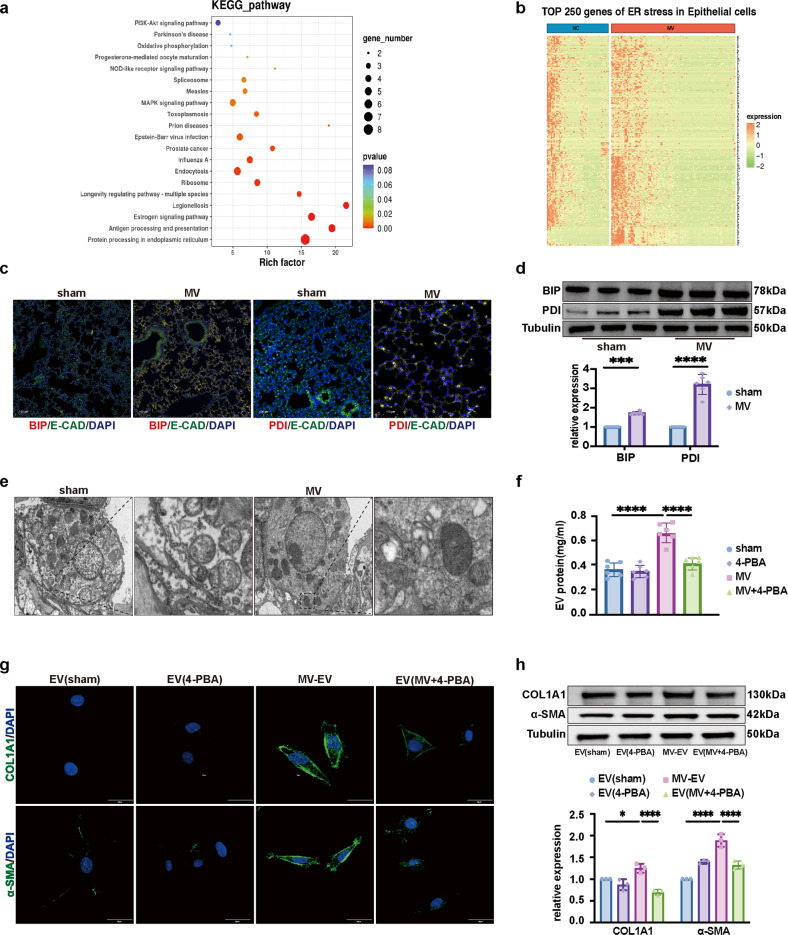
MV-induced ER stress-mediated fibrotic MV-EV release to promote lung fibroblast activation. **a** KEGG enrichment analysis of the top 250 DEGs in alveolar epithelial cells. **b** Heatmap showing the upregulated and downregulated genes related to ER stress in alveolar epithelial cells. **c** Lung tissues were stained with fluorophore-labeled antibodies against the ER stress markers BIP and PDI (Alexa Fluor 594, red) and the epithelial cell marker E-cadherin (E-CAD) (Alexa Fluor 488, green). 4’,6-Diamidino-2-phenylindole (DAPI) stain was used to detect nuclei (blue). Original magnification ×200. Scale bars correspond to 100 μm (*n* = 6). **d** Protein expression of BIP and PDI in lung homogenates was determined by WB. Relative intensity of the protein bands of BIP and PDI compared to that of Tubulin as determined by densitometry is displayed in bar graphs (*n* = 6). **e** Representative TEM images of the ER in alveolar epithelial cells in lung tissues (*n* = 6). **f** EVs isolated from BALF were quantified by protein BCA (*n* = 6). **g** MRC-5 cells were stained with fluorophore-labeled antibodies against COL1A1 and α-SMA (Alexa Fluor 488, green). 4’,6-Diamidino-2-phenylindole (DAPI) stain was used to detect nuclei (blue). Original magnification ×800. Scale bars correspond to 20 μm (*n* = 3). **h** Protein expression of COL1A1 and α-SMA in MRC-5 cells was determined by WB. Relative intensity of the protein bands of COL1A1 and α-SMA compared with that of Tubulin as determined by densitometry is displayed in bar graphs (*n* = 3). The data are expressed as the means ± SEMs. *p < 0.05, **p < 0.01, ***p < 0.001, ****p < 0.001.

Single-cell RNA-sequencing analysis was used to explore the mechanisms involved in MVPF. A UMAP plot of all cell types is shown in Supplementary Fig. 2a, and cluster marker genes are shown in Supplementary Fig. 2b. Supplementary Fig. 2c shows the top 250 DEGs in epithelial cells, and a pathway enrichment analysis indicated that the response to ER stress signaling pathway and ASK1 pathway were significantly changed (Fig. 5a). Notably, ER stress-related gene expression was upregulated in epithelial cells after MV (Fig. 5b).

Further validation of the single-cell RNA-Seq data was achieved by measuring the expression of BIP and PDI in MVPF lung tissue. The increased amounts of BIP+/E-cad+ and PDI+/E-cad+ epithelial cells were observed in the MV group (Fig. 5c, Supplementary Fig. 3a), and the increased protein levels of BIP and PDI in pulmonary tissue were detected by WB (Fig. 5d). Additionally, the ultrastructure of the ER was observed by TEM (Fig. 5e). Moreover, an increase in MV-EV release was observed in the BALF after MV (Fig. 5f).

After MRC-5 cells were treated with MV-EVs (50 μg of EV protein/sample), the protein levels of COL1A1 and α-SMA were increased significantly (Fig. 5h), and an increase in the percentage of α-SMA+/COL1A1+ cells was observed via immunofluorescence assay (Fig. 5g, Supplementary Fig. 3b). These processes were suppressed in mice by pretreatment with 4-PBA before MV (Fig. 5f–h).

Taken together, these data suggested that MV-induced ER stress, which mediated an increase in the MV-EV release to promote lung fibroblast activation.

### MV-induced ASK1 activation mediated ER stress and fibrotic MV-EV release to promote lung fibroblast activation

**Fig. 6:**
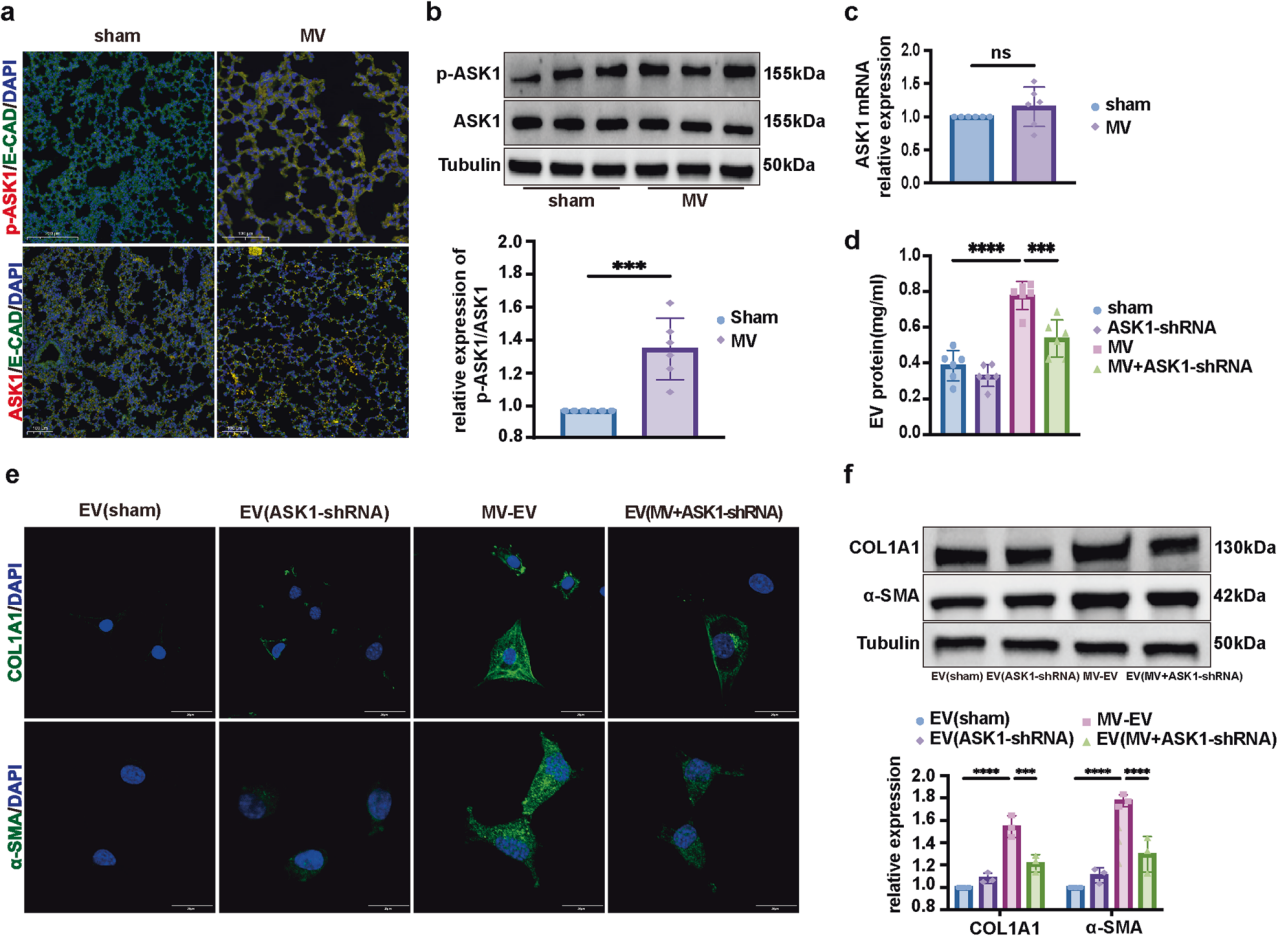
MV-induced ASK1 activation mediated ER stress and fibrotic MV-EV release to promote lung fibroblast activation. **a** Lung tissues were stained with fluorophore-labeled antibodies against ASK1 and p-ASK1 (Alexa Fluor 594, red) and the epithelial cell marker E-cadherin (E-CAD) (Alexa Fluor 488, green). 4’,6-Diamidino-2-phenylindole (DAPI) stain was used to detect nuclei (blue). Original magnification ×200. Scale bars correspond to 100 μm (*n* = 6). **b** ASK1 and p-ASK1 protein expression in lung homogenates was determined by WB. Relative intensity of the protein bands of ASK1 and p-ASK1 compared to that of Tubulin as determined by densitometry is displayed in bar graphs (*n* = 6). **c** ASK1 mRNA expression in lung homogenates was determined by qRT‒PCR. The relative mRNA expression of ASK1 compared to that of GAPDH is displayed in bar graphs (*n* = 6). **d** EVs isolated from BALF were quantified by protein BCA (*n* = 6). **e** MRC-5 cells were stained with fluorophore-labeled antibodies against COL1A1 and α-SMA (Alexa Fluor 488, green). 4’,6-Diamidino-2-phenylindole (DAPI) stain was used to detect nuclei (blue). Original magnification ×800. Scale bars correspond to 20 μm (*n* = 3). **f** Protein expression of COL1A1 and α-SMA in MRC-5 cells was determined by WB. Relative intensity of the protein bands of COL1A1 and α-SMA compared with that of Tubulin as determined by densitometry is displayed in bar graphs (*n* = 3). The data are expressed as the means ± SEMs. *p < 0.05, **p < 0.01, ***p < 0.001, ****p < 0.001.

MV significantly increased the percentage of p-ASK1+/E-cad+ epithelial cells, while the percentage of ASK1+/E-cad+ epithelial cells was not changed in pulmonary tissue compared with percentage in the sham group (Fig. 6a, Supplementary Fig. 3c). Moreover, the protein expression of p-ASK1 was upregulated, while the levels of ASK1 were unchanged in the pulmonary tissue of the MV group (Fig. 6b). In contrast, qRT‒PCR indicated that the mRNA level of ASK1 was not changed in the MV group (Fig. 6c).

We next sought to determine whether ASK1 activation was involved in MV-induced ER stress and MV-EV release. ASK1-shRNA-AAV was used to infect mouse pulmonary tissue via intratracheal injection to knock down the ASK1 gene and cause a decrease in ASK1 level in the MV group (Supplementary Fig. 3d). As shown in Fig. 6d, ASK1-shRNA reduced the EV release in the BALF compared with that in the MV group. After MRC-5 cells were treated with MV-EVs (50 μg of EV protein/sample), upregulation of COL1A1 and α-SMA was detected by WB (Fig. 6f). This was confirmed by immunofluorescence assay (Fig. 6e, Supplementary Fig. 3e). As shown in Fig. 6d–f, MV-EV-induced MRC-5 cell activation was alleviated in the ASK1-shRNA group.

Therefore, we speculated that MV activated the ASK1-ER stress pathway to increase the MV-EV release and to mediate the activation of lung fibroblasts.

### Inhibiting ER stress contributed to the alleviation of MV-induced pulmonary fibrosis

**Fig. 7:**
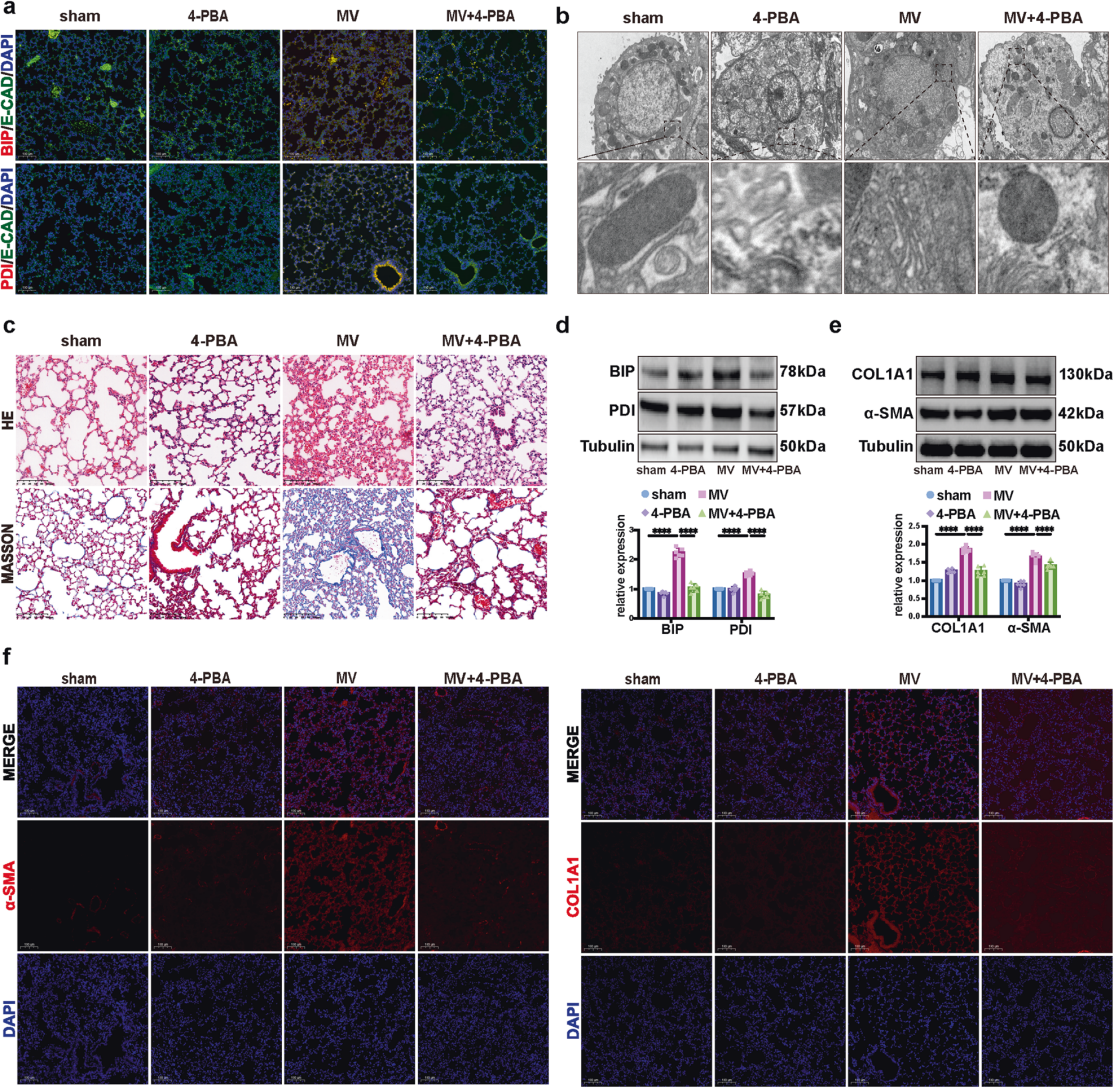
Inhibiting ER stress contributed to the alleviation of MV-induced pulmonary fibrosis. **a** Lung tissues were stained with fluorophore-labeled antibodies against the ER stress markers BIP and PDI (Alexa Fluor 594, red) and the epithelial cell marker E-cadherin (E-CAD) (Alexa Fluor 488, green). 4’,6-Diamidino-2-phenylindole (DAPI) stain was used to detect nuclei (blue). Original magnification ×200. Scale bars correspond to 100 μm (*n* = 6). **b** Representative TEM images of ER in alveolar epithelial cells of lung tissues (*n* = 6). **c** Lung injury was assessed by hematoxylin and eosin staining. Collagen deposition was assessed with Masson’s trichrome staining. **d** Protein expression of BIP and PDI in lung homogenates was determined by WB. Relative intensity of the protein bands of BIP and PDI compared with that of Tubulin as determined by densitometry is displayed in bar graphs (*n* = 6). **e** Protein expression of COL1A1 and α-SMA in lung homogenates was determined by WB. Relative intensity of the protein bands of COL1A1 and α-SMA compared with that of Tubulin as determined by densitometry is displayed in bar graphs (*n* = 6). **f** Lung tissues were stained with fluorophore-labeled antibodies against COL1A1 and α-SMA (Alexa Fluor 594, red). 4’,6-Diamidino-2-phenylindole (DAPI) stain was used to detect nuclei (blue). Original magnification ×200. Scale bars correspond to 100 μm (*n* = 6). The data are expressed as the means ± SEMs. *p < 0.05, **p < 0.01, ***p < 0.001, ****p < 0.001.

To investigate whether inhibition of ER stress in vivo affects MVPF, mice were intraperitoneally pretreated with 4-PBA. Immunofluorescence staining showed an increased percentage of BIP+/E-cad+ and PDI+/E-cad+ epithelial cells after 4-PBA pretreatment (Fig. 7a, Supplementary Fig. 4a). As shown in Fig. 7b, c, ER ultrastructure changes and pulmonary histopathology were attenuated by 4-PBA pretreatment. WB showed upregulated protein levels of BIP and PDI in pulmonary tissue (Fig. 7d). Consistent with the upregulation of COL1A1 and α-SMA in pulmonary tissue (Fig. 7e), immunofluorescence staining showed that MV significantly increased the percentage of α-SMA+ and COL1A1+ cells among total cells (Fig. 7f). Furthermore, these responses were alleviated by pretreatment with 4-PBA (Fig. 7a–f).

Therefore, we speculated that inhibiting ER stress contributed to the alleviation of MV-induced pulmonary fibrosis.

### Inhibiting ASK1 activation contributed to the alleviation of ER stress and MV-induced pulmonary fibrosis

**Fig. 8:**
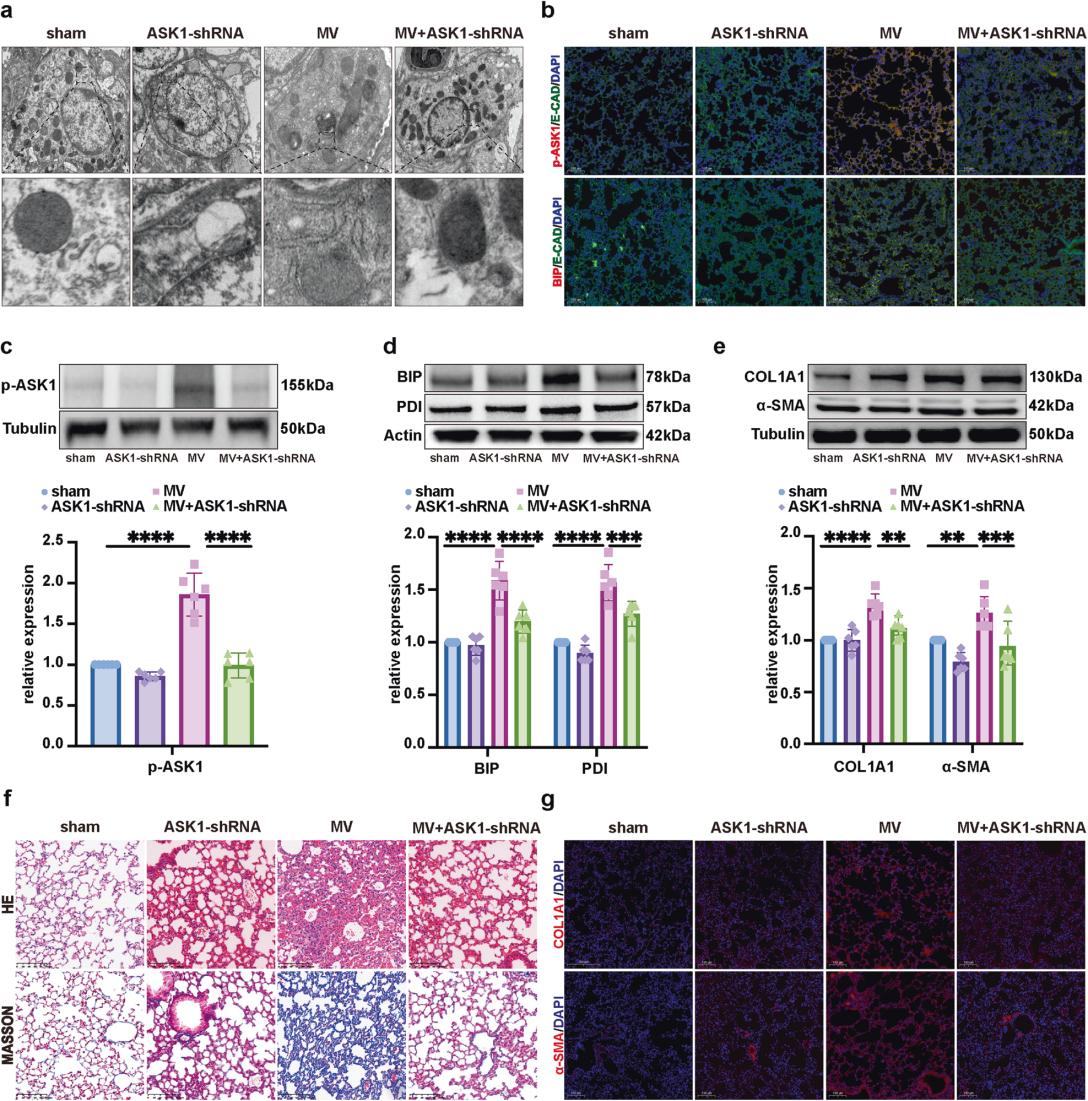
Inhibiting ASK1 activation contributed to the alleviation of ER stress and MV-induced pulmonary fibrosis. **a** Representative TEM images of ER in alveolar epithelial cells of lung tissues (*n* = 6). **b** Lung tissues were stained with fluorophore-labeled antibodies against p-ASK1 and BIP (Alexa Fluor 594, red) and the epithelial cell marker E-cadherin (E-CAD) (Alexa Fluor 488, green). 4’,6-diamidino-2-phenylindole (DAPI) stain was used to detect nuclei (blue). Original magnification ×200. Scale bars correspond to 100 μm (*n* = 6). **c** Protein expression of p-ASK1 in lung homogenates was determined by WB. Bar graphs display the relative intensity of the protein bands of p-ASK1 compared to that of Tubulin as determined by densitometry (*n* = 6). **d** Protein expression of BIP and PDI in lung homogenates was determined by WB. Bar graphs display the relative intensity of the protein bands of BIP and PDI compared to that of Actin as determined by densitometry (*n* = 6). **e** Protein expression of COL1A1 and α-SMA in lung homogenates was determined by WB. Bar graphs display the relative intensity of the protein bands of COL1A1 and α-SMA compared to that of Tubulin as determined by densitometry (*n* = 6). **f** Lung injury was assessed by hematoxylin and eosin staining. Collagen deposition was assessed with Masson’s trichrome staining (*n* = 6). **g** Lung tissues were stained with fluorophore-labeled antibodies against COL1A1 and α-SMA (Alexa Fluor 594, red). 4’,6-Diamidino-2-phenylindole (DAPI) stain was used to detect nuclei (blue). Original magnification ×200. Scale bars correspond to 100 μm (*n* = 6). The data are expressed as the means ± SEMs. *p < 0.05, **p < 0.01, ***p < 0.001, ****p < 0.001.

Recent studies have shown that ER stress is related to the activation of ASK1^12^, which is closely related to liver or kidney fibrosis^13,14^. Activation of ASK1 has also been involved in the process of VILI^15^. To assess whether ASK1 activation is essential for MV-induced ER stress and pulmonary fibrosis, we performed WB, histopathology and immunofluorescence assay, and TEM to analyze pulmonary samples. We found that the ER ultrastructure was changed in epithelial cells after MV, as shown in Fig. 8a. MV significantly increased the percentage of p-ASK1+/E-cad+, BIP+/E-cad+ (Fig. 8b, Supplementary Fig. 4b, c), and PDI+/E-cad+ (Supplementary Fig. 4d) epithelial cells. In contrast, the percentage of ASK1+/E-cad+ epithelial cells was not changed in pulmonary tissue compared with that in the sham group (Supplementary Fig. 4e). WB showed upregulated protein expression of p-ASK1, BIP, and PDI in pulmonary tissue (Fig. 8c, d). Moreover, the expression of COL1A1 and α-SMA was increased in pulmonary tissue (Fig. 8e). Consistent with the changes in pulmonary histopathology (Fig. 8f), immunofluorescence staining showed that MV significantly increased the percentage of α-SMA+ and COL1A1+ among total cells (Fig. 8g, Supplementary Fig. 4f). Notably, ASK1 downregulation alleviated all these responses in pulmonary tissue (Fig. 8a–g). These findings suggested that inhibiting ASK1 activation contributed to the alleviation of ER stress and MV-induced pulmonary fibrosis.

## Discussion

MV is an effective treatment option for patients with respiratory failure. However, MV can lead to VILI, MVPF, and other adverse effects. Previous clinical studies showed that among ARDS patients who received MV for >12 days, the incidence of PF was as high as 63%^16^, and the mortality of these patients was as high as 57%^17^. The mechanisms of MVPF have been investigated using rodent models exposed to high tidal volume ventilation^18,19^. However, the mechanisms underlying MVPF are still unclear. In this study, we found that ASK1-ER stress-mediated fibrotic-EV release plays an essential role in the interaction of alveolar epithelial cells and lung fibroblasts, which might promote mechanical ventilation-induced pulmonary fibrosis.

ER stress leads to activation of the unfolded protein response triggered by the misfolding and aggregation of unfolded proteins within the ER lumen and calcium imbalance under stress conditions. In recent years, ER stress has been recognized as an important pathogenic mechanism for several types of fibrosis^20^, but the mechanism that regulates ER stress in MVPF has not been fully investigated. There is a link between ER stress and the UPR and PF, which is mediated through the regulation of alveolar epithelial cell death, the epithelial-mesenchymal transition, differentiation of myofibroblasts, and polarization of M2 macrophages^21^. Importantly, several studies have reported that an increase in the ER stress response can activate PI3K/AKT, hypoxia, and the HIF-1 and ATF3/PINK1 signaling pathways^22–24^. A previous study focused on the effect of ER stress on a single pulmonary cell type. However, the present study revealed that ER stress in alveolar epithelial cells promoted the release of fibrotic-EV, which led to the activation of lung fibroblasts and the onset of pulmonary fibrosis. Furthermore, the present study revealed that the inhibition of ER stress by 4-PBA alleviated ER stress and MVPF. Therefore, our findings suggest that ER stress-induced fibrotic-EV release may be involved in the pathogenesis of MVPF.

**Fig. 9:**
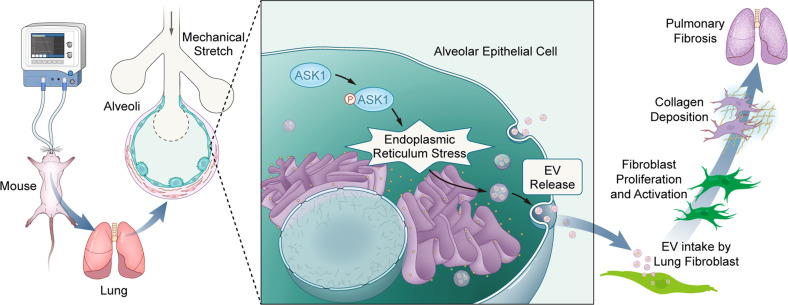
Diagram showing ASK1-ER stress-mediated fibrotic-EV release contributing to MV-induced pulmonary fibrosis.

ASK1 is a member of the kinase’s family of mitogen-activated protein kinases, which plays key roles in apoptosis induced by cytokines and stress and is closely related to liver or kidney fibrosis^13,14^. ASK1 activation has been found to be involved in the pathogenesis of VILI, and inhibition of ASK1 can inhibit the acute inflammatory response of VILI^15^. Furthermore, recent studies have shown that ASK1 leads to liver fibrosis by increasing EV release through ER stress^7^. Consistent with these findings, we found that ASK1 was involved in MVPF and enhanced ER stress-dependent fibrotic-EV release. Specifically, inhibition of ASK1 mitigated ER stress-dependent fibrotic-EV release and MVPF, which indicated that ASK1 acts as a mechanical sensor that accelerates ER stress-dependent fibrotic-EV release to induce PF (Fig. 9).

It has long been suspected that fibroblasts and structural epithelial cells in the lungs communicate via messengers. To this end, cytokines are thought to play a particularly important role in transmitting stress/inflammatory signals between cells^25,26^. However, the treatment of ARDS/ALI with the aim of inhibiting inflammatory cytokine action has not been proven to be very effective^27^. It is likely that EVs serve as a kind of ‘shelter’s for signaling proteins, thereby preventing degradation in the ECM, and thus serves as a means of transmit various fibrotic signals. Our results demonstrate that fibrotic-EVs contribute to fibroblast activation and the initiation of pulmonary fibrosis during MV. In our upcoming study, we will focus on the mechanism of MV-EVs in fibroblast activation and the initiation of pulmonary fibrosis. As a limitation of this study, not all the protein contents of these fibrotic-EVs derived from alveolar epithelial cells were identified. Therefore, comprehensive proteomics of EVs secreted from all cell types is planned to address this limitation.

The present study demonstrates that ASK1-ER stress pathway-mediated fibrotic-EV release from alveolar epithelial cells contributes to fibroblast activation and the onset of pulmonary fibrosis during MV. The inhibition of EV release targeting the ASK1-ER stress pathway may be a promising treatment strategy for MVPF.

## Supplementary information

Supplemental material
