# Supplementary material for: ASK1-ER stress pathway-mediated fibrotic-EV release contributes to the interaction of alveolar epithelial cells and lung fibroblasts to promote mechanical ventilation-induced pulmonary fibrosis
Source: Exp Mol Med. 2022 Dec 6;54(12):2162–74. doi: 10.1038/s12276-022-00901-1 (PMC9734805; doi:10.1038/s12276-022-00901-1)
Supplement: Supplementary file 1 — Supplemental material [file 12276_2022_901_MOESM1_ESM.pdf]

Supplementary Fig. 1

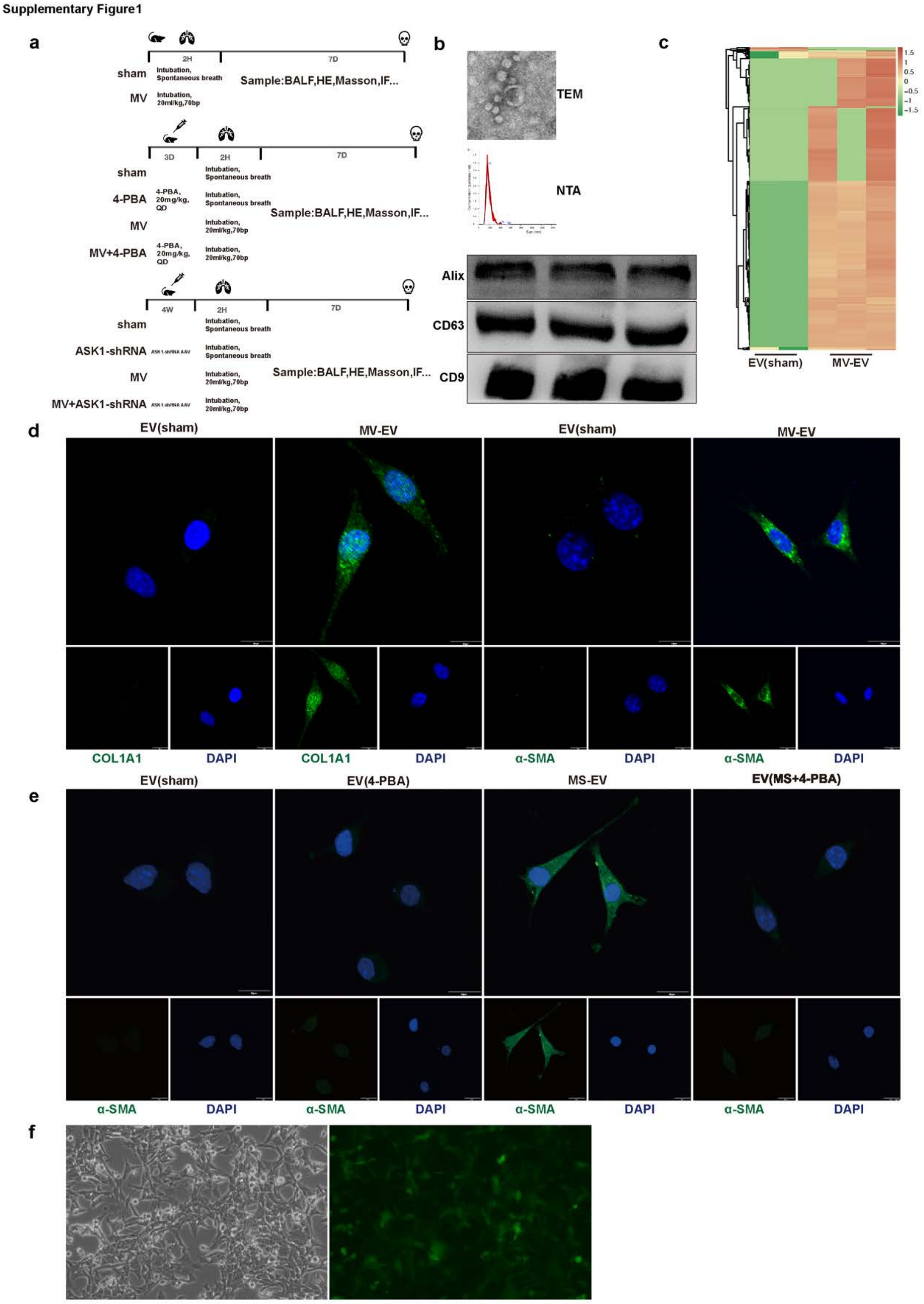

Supplementary Fig. 2

Supplementary Figure2

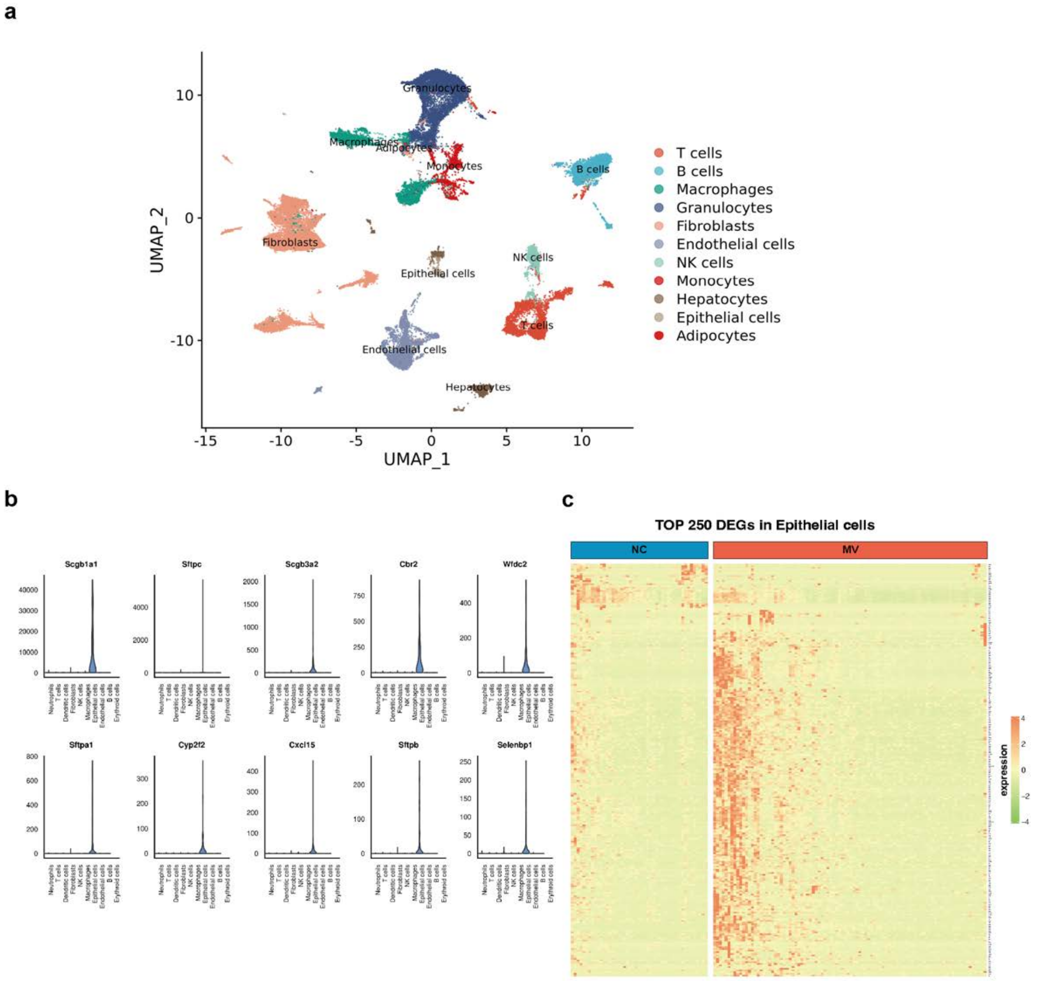

Supplementary Fig. 3

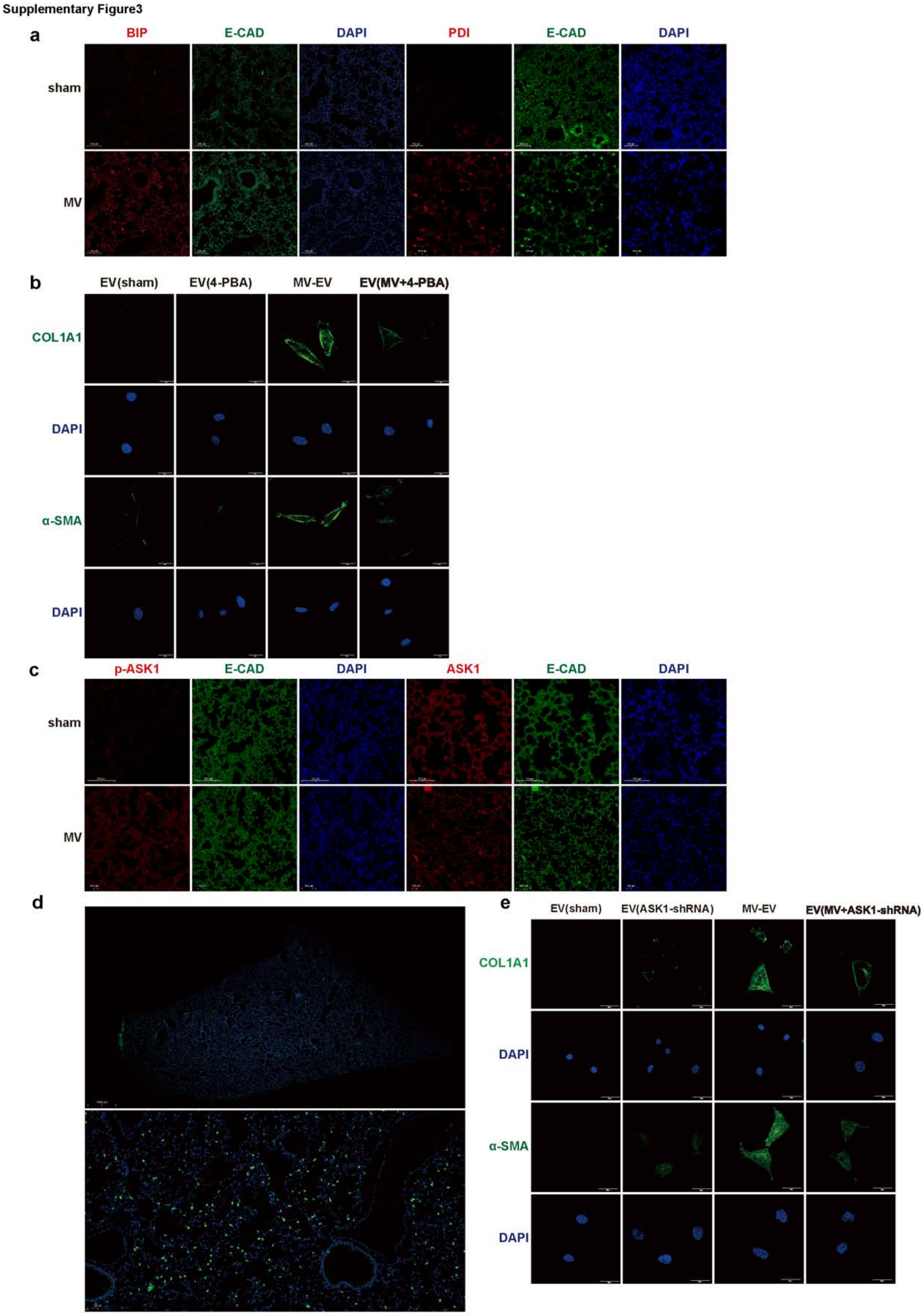

Supplementary Fig. 4

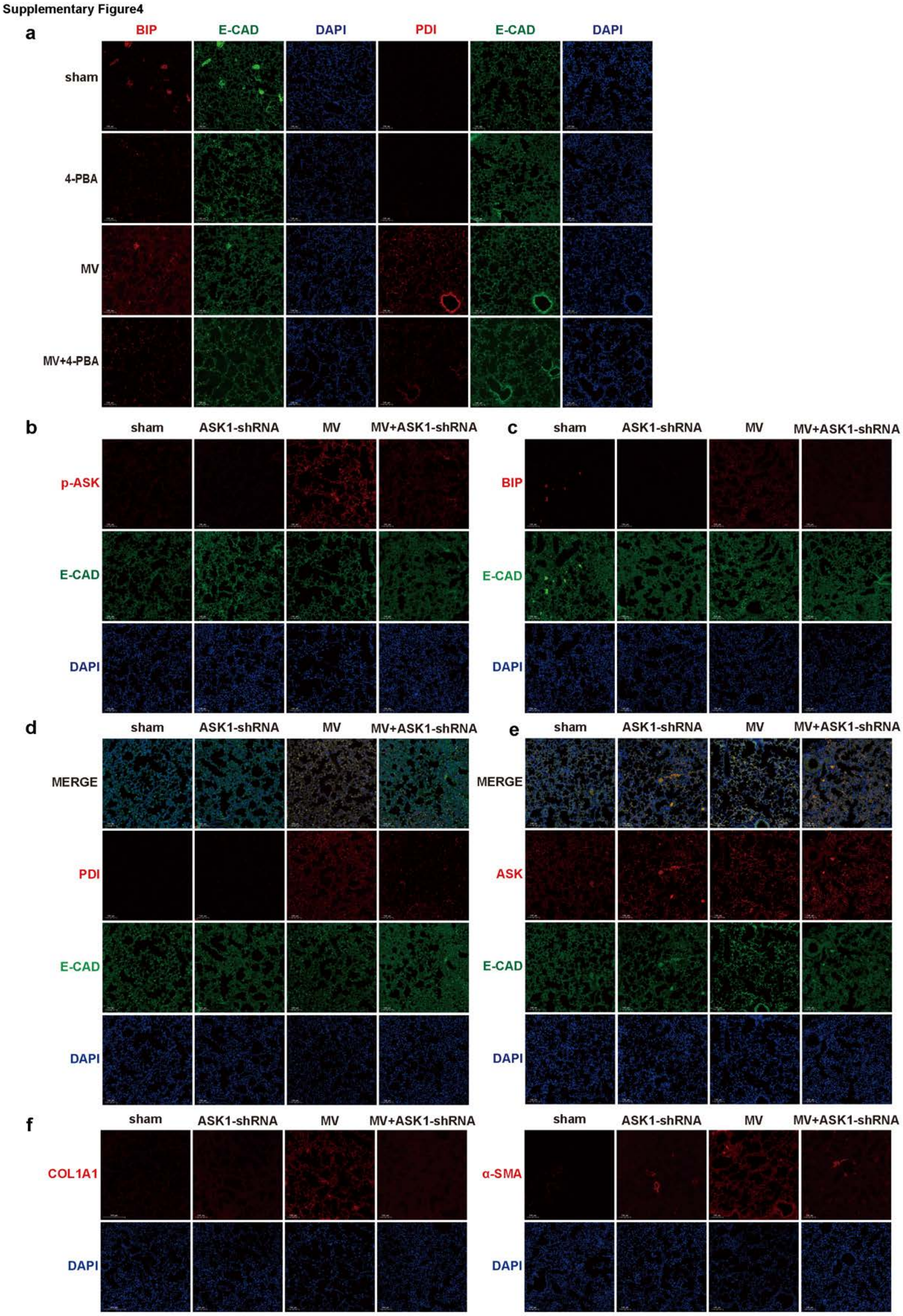

Supplementary Table 1. Primary antibodies used in this study

Antigens	Manufacturer	Catalog Number	Application
ASK1	Cell signaling technology	#8662	1:1000 for WB
ASK1	NOVUS	NB100-81788	1:100 for IF
p-ASK1	Invitrogen	PA5-105027	1:1000 for WB; 1:100 for IF
E-cadherin	Abcam	ab231303	1:100 for IF
BIP	Cell signaling technology	#3177S	1:1000 for WB; 1:100 for IF
PDI	Cell signaling technology	#3501S	1:1000 for WB; 1:100 for IF
COL1A1	Abcam	ab138492	1:1000 for WB; 1:100 for IF
α-SMA	Abcam	ab124964	1:1000 for WB; 1:100 for IF
Actin	Cell signaling technology	#4979S	1:1000 for WB
Tubulin	Cell signaling technology	#2148S	1:1000 for WB
